# Ultrahypofractionated Versus Normofractionated Preoperative Radiotherapy for Soft Tissue Sarcoma: A Multicenter, Prospective Real-World-Time Phase 2 Clinical Trial

**DOI:** 10.3390/cancers16234063

**Published:** 2024-12-04

**Authors:** Philip Heesen, Michele Di Lonardo, Olga Ciobanu-Caraus, Georg Schelling, Daniel Zwahlen, Beata Bode-Lesniewska, Christoph Glanzmann, Gabriela Studer, Bruno Fuchs

**Affiliations:** 1 Medical Faculty, University of Zurich, 8032 Zurich, Switzerland; philip.heesen@uzh.ch; 2 Sarcoma Service, Department of Orthopedics and Trauma, Sarcoma Center, Radiation Oncology, LUKS University Hospital, 6000 Luzern, Switzerlandgeorg.schelling@luks.ch (G.S.); gabriela.studer@luks.ch (G.S.); 3 Faculty of Medicine, Medical University of Vienna, Spitalgasse 23, 1090 Vienna, Austria; 4 Faculty of Health Sciences & Medicine, University Lucerne, Frohburgstrasse 3, 6002 Luzern, Switzerland; 5 Sarcoma Service, Klinik für Orthopädie und Traumatologie, Radiation Oncology, Sarcoma Center, Kantonsspital Winterthur, 8400 Winterthur, Switzerland

**Keywords:** soft tissue sarcoma, ultrahypofractionated radiotherapy (uhRT), normofractionated radiotherapy (nRT), preoperative radiation therapy, clinical trial, wound complications, local control rate

## Abstract

**Background/Objectives:** The historically most commonly used preoperative radiotherapy regimen for soft tissue sarcomas (STSs) consists of 50 Gray (Gy) delivered in 25 fractions over 5 weeks, achieving excellent local control, but with significant challenges due to prolonged treatment duration and early side effects. Reducing therapy duration while maintaining optimal local and distant control would be highly beneficial for patients. We aimed to investigate the outcome of an ultrahypofractionated radiotherapy (uhRT) regimen which may represent a shorter and more patient-friendly alternative. **Methods:** This multi-center, open-label, phase 2 clinical trial with a clustered cohort design was conducted within the Swiss Sarcoma Network (SSN). Adult patients (aged ≥ 18 years) with STS of the extremities or superficial trunk and an Eastern Cooperative Oncology Group (ECOG) performance status of 0–3 were included. Participants were assigned to either normofractionated radiotherapy (nRT) at 50 Gy in 25 fractions or uhRT at 25 Gy in 5 fractions. Data were collected prospectively in real-world-time clinical settings. The primary outcome was local recurrence-free survival (LRFS), with overall survival (OS) and wound complications as secondary outcomes. **Results:** Between March 2020 and October 2023, 138 patients were included in the study; 74 received nRT and 64 received uhRT. The median follow-up times were 2.2 years for uhRT and 3.6 years for nRT. The LRFS rates at 1 year were 97.0% for nRT and 94.8% for uhRT (*p* = 0.57). The two-year LRFS rates were 91.9% and 94.8%, respectively (*p* = 0.57). The one- and two-year OS rates were 97.1%/86.3% and 98.2%/88.8%, respectively (*p* = 0.72). The wound complication rate was comparable between the nRT (12.0%) and uhRT (12.5%) groups (*p* = 0.99). **Conclusions:** UhRT for STSs offers an effective and safe alternative to traditional nRT, with comparable early LRFS, OS and wound complication rates. Given the two-year median follow-up, which is critical for evaluating local recurrence, uhRT shows promise as a shorter and more convenient treatment regimen. UhRT may be a safe and effective alternative treatment option to traditional nRT.

## Simple Summary

Soft tissue sarcomas are rare cancers that are often treated with a combination of surgery and preoperative radiotherapy to reduce the risk of cancer returning. Traditionally, this radiotherapy is delivered over five weeks, which can be challenging for patients due to the long treatment time and potential side effects. This study investigates a shorter, one-week radiotherapy option to determine if it provides similar results in terms of cancer control, survival and wound healing. By comparing these two treatment schedules, we aim to explore whether the shorter approach can offer a safe and effective alternative that might reduce the burden on patients and healthcare systems. If successful, this shorter treatment could improve patient convenience and resource efficiency, offering a new option in sarcoma care that aligns with modern goals for patient-centered, efficient cancer treatments.

## 1. Introduction

Soft tissue sarcomas (STSs) are a rare, heterogeneous group of malignant tumors originating in the connective tissues, including muscles, fat and nerves, presenting diverse histological types and aggressiveness [1]. Standard therapy involves surgical resection supplemented by radiotherapy, which has been shown to significantly reduce the risk of local recurrence [2,3]. Radiotherapy should be administered preoperatively, aiming to sterilize adjacent tissues and thereby improve local control.

Advancements in radiotherapy technologies have substantially improved the precision and efficacy of treatments for STSs. Techniques such as IMRT (Intensity-Modulated Radiotherapy) and VMAT (Volumetric Modulated Arc Therapy) have optimized the delivery of radiation by enabling highly conformal dose distributions that closely match the planning target volume (PTV)’s three-dimensional shape [4]. This allows for maximized radiation delivery to the PTV while better sparing surrounding healthy tissues, which is crucial in reducing the incidence of side effects such as would complications.

Radiation dose fractionation has evolved alongside these technological advancements. The currently most used normofractionated radiotherapy (nRT) administers approximately 2 Gy per fraction over several weeks [5,6]. Recently, hypofractionated (hRT) and ultrahypofractionated (uhRT) regimens have emerged as promising alternatives [7,8,9,10,11]. UhRT delivers higher doses per fraction in fewer sessions over one to two weeks. This approach is based on radiobiological evidence suggesting that STSs have a low α/β ratio, resulting in higher sensitivity to larger radiation doses per fraction. Hypofractionated radiotherapy has been shown to effectively induce immunogenic tumor cell death, releasing tumor-associated antigens and damage-associated molecular patterns. Furthermore, due to an increased expression of Major Histocompatibility Complex I, antigen recognition is promoted. Another potential mechanism of action of hypofractionation is the induction of bystander cells [12,13]. Due to the reduced treatment duration, uhRT represents a more efficient and potentially less burdensome alternative therapeutic regimen for the treatment of STSs. Additionally, uhRT could enhance resource utilization and decrease healthcare costs.

Several studies have shown that uhRT can achieve comparable outcomes to nRT [8,10,11,14,15,16,17,18,19,20,21,22,23,24]. Research at MD Anderson Cancer Center demonstrated the safety and convenience of a three-week mild hypofractionated preoperative regimen without increasing major wound complications [25].

However, comprehensive real-world-time (RWT) data comparing uhRT and nRT in STSs are lacking, potentially hindering the broader adoption of uhRT in clinical practice. This study aimed to compare the efficacy of uhRT versus nRT in treating STSs.

## 2. Materials and Methods

### 2.1. The Study Design and Participants

This study was a multi-center, prospective real-world-time, open-label, phase II clinical trial with a clustered cohort design (cluster randomization). Treatment decisions were based on institutional preference and influenced by external factors such as the COVID-19 pandemic. One center exclusively administered uhRT since March 2020 [26,27]. The inclusion criteria were as follows: adults (aged ≥ 18 years) with STS of the extremities or superficial trunk with an estimated life expectancy of at least six months who had received preoperative radiotherapy; an ECOG performance status of 0 to 3; a histological diagnosis of intermediate-to-high-grade STS or high-risk low-grade STS according to the FNCLCC classification; and the technical possibility of gross-total macroscopic resection a determined by the interdisciplinary tumor board MDT/SB. Patients were excluded if they had received radiotherapy post-operatively or for palliative purposes.

An experienced specialized sarcoma surgeon (BF) performed all operations in both treatment groups. Indications of preoperative radiotherapy were made upon the recommendations of the same interdisciplinary tumor board. All uhRT treatment delineations of the cases were performed/supervised by the same radiation oncologists (GS, CG). This study complies with the principles of the Declaration of Helsinki and was approved by the Kantonale Ethikkomission Zurich, Switzerland (BASEC-Nr. 2019-01107 on 24 August 2021; registered on clinicaltrials.gov under NCT04300257). All participants provided written informed consent.

### 2.2. Treatment

Patients were treated with either nRT (50 Gy in 25 fractions in 5 weeks) or uhRT (25 Gy in 5 fractions in 1 week). The gross tumor volume was initially defined based on preoperative co-registered MRI and adjusted for peritumoral edema. The PTV (planning target volume) incorporated an omnidirectional margin of 1.5 cm and a longitudinal margin of 3–4 cm. Individual manual editing of the PTV was routinely performed to spare non-affected tissue, particularly bone and non-involved skin. If tumors were adjacent to the skin or if a biopsy was performed prior to radiotherapy, bolus material of 1 cm thickness was applied to ensure adequate dose delivery. Radiotherapy was administered using Volumetric Modulated Arc Therapy or Intensity-Modulated Radiation Therapy, complemented by Image-Guided Radiotherapy.

### 2.3. Data Collection

Data were prospectively collected using Sarconnector^®^/SHAPEHUB^®^ (v1.1), a real-world-time digital interoperable platform shared across all participating institutions of the Swiss Sarcoma Network [26,27]. This platform collects demographic details, clinical characteristics, treatment specifics and outcomes in a structured and harmonized manner.

### 2.4. Outcome Measures

The primary outcome of this study was local recurrence-free survival (LRFS). The secondary outcomes were overall survival (OS) and wound complications within 120 days after surgery. Wound complications included revision surgery, vacuum-assisted closure and major infections as per the Canadian NCIC SR2 trial standards [5,25]. Early/intermediate disease control (Table 1) was also assessed according to clinical and imaging follow-ups.

Regular follow-up assessments were scheduled every 3 months during the first 2–3 years, and subsequently every 6 months. These evaluations involved MRI scans of the primary tumor site and CT scans of the thorax to detect local recurrence and metastasis. For patients with metastatic disease, imaging and follow-up assessments were scheduled according to the recommendations of the interdisciplinary tumor boards.

### 2.5. Statistical Analysis

Continuous variables are presented as the median and interquartile range (IQR), and categorical variables as the frequency and percentage. Comparisons between nRT and uhRT were conducted using Pearson’s Chi-squared test for categorical data and the Wilcoxon rank-sum test for continuous data. The predicted overall survival was estimated using the Sarculator [28]. Variables that were needed to calculate the predicted survival using the Sarculator were not available for all patients. In these cases (n = 35), the predicted survival was calculated using Persarc [29]. Survival outcomes were analyzed using the Kaplan–Meier method. Differences in survival times over the whole study period were assessed using the log-rank test, while differences in survival probabilities at certain time points were tested using a Z-test. The hazard ratios (HRs) and 95% Confidence Intervals (CIs) for OS and LRFS were estimated using Cox proportional hazards regression, adjusted for the predicted overall survival as estimated by the Sarculator or Persarc [28,29]. The odds ratios (ORs) and 95% CIs for wound revision rates were calculated using logistic regression, adjusted for the predicted overall survival. All *p*-values were two-tailed, with an alpha value of 0.05.

### 2.6. Quality Control

Quality control measures to ensure the accuracy and reliability of data were implemented within the Sarconnector^®^/SHAPEHUB^®^ [26,27]. Validation checks for completeness and correctness were systematically performed. Outliers and anomalies were reviewed by clinical experts to ensure data validity.

## 3. Results

### 3.1. Patients Characteristics

Of 138 patients undergoing preoperative radiation therapy for STSs, 74 (53.6%) received nRT, while 64 (46.4%) were treated with uhRT. The patient characteristics are presented in Table 2. A higher proportion of patients with FNCLCC high-grade (G3) tumors were treated under the uhRT protocol compared to the nRT protocol (70% vs. 48%, *p* = 0.007). The predicted 5-year overall survival rates as determined by the Sarculator and Persarc prognostic tools did not differ significantly between the groups (*p* = 0.10) [29,30,31,32]. Supplementary Table S1 presents the diagnoses of the included patients stratified by therapy group.

### 3.2. Local Recurrence-Free Survival

**Figure 1:**
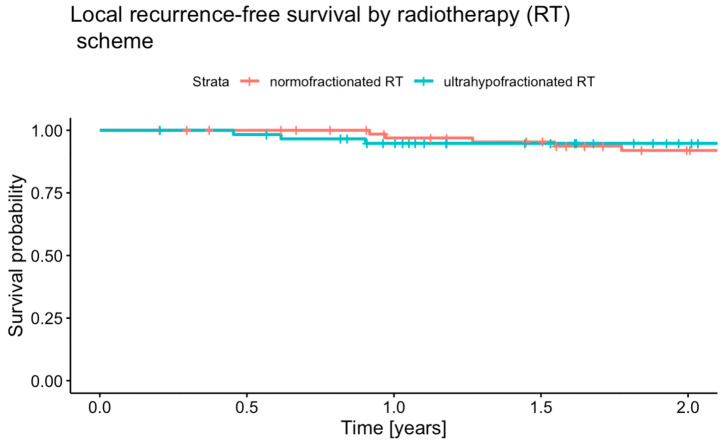
Kaplan–Meier plot of local recurrence-free survival stratified by therapy group.

The median follow-up time for LRFS was 2.2 years (95% CI 2.0–2.5) in the uhRT group and 3.6 years (95% CI 2.8–4.3) in the nRT group. Local recurrence occurred in 8 (10.8%) of 74 patients in the nRT group, and in 4 (6.3%) of 64 patients in the uhRT group. The LRFS estimates at one and two years are shown in Figure 1. The one-year LRFS rates were 97.0% (95% CI 92.9–100.0) in the nRT group and 94.8% (95% CI 89.2–100.0) in the uhRT group (*p* = 0.57). The two-year LRFS rates were 91.9% (95% CI 85.4–99.0) for nRT and 94.8% (95% CI 89.2–100.0) for uhRT (*p* = 0.57). The log-rank *p*-value was 0.93. The HR for LR following uhRT relative to nRT was 0.54 (95% CI 0.11–2.76, *p* = 0.46).

### 3.3. Overall Survival

**Figure 2:**
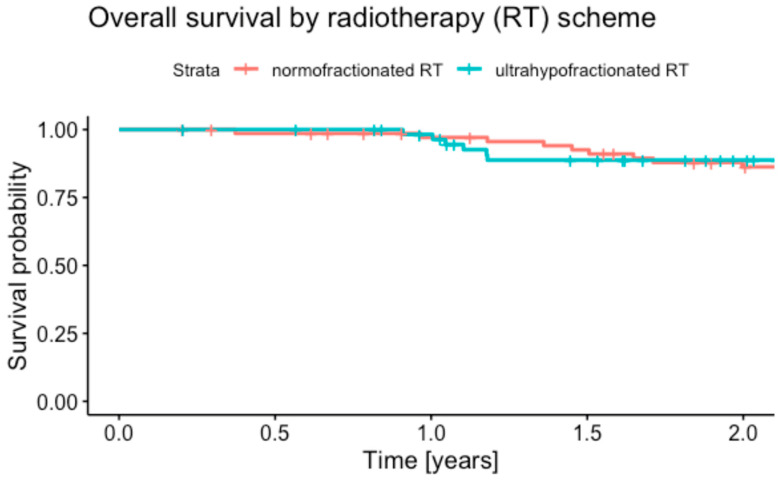
Kaplan–Meier plot of overall survival stratified by therapy group.

The median follow-up times for OS were 2.3 years (95% CI 2.1–2.8) in the uhRT group, and 4.2 years (95% CI 3.4–4.8) in the nRT group. Deaths from any cause occurred in 19 (25.7%) of 74 patients in the nRT group, and 11 (17.2%) of 64 patients in the uhRT group. The one-year OS rates were 97.1% (95% CI 93.2–100.0) in the nRT group and 98.2% (95% CI 94.8–100.0) in the uhRT group (Figure 2, *p* = 0.69). At two years, the OS rates were 86.3% (95% CI 78.3–95.0) for nRT and 88.8% (95% CI 80.7–97.7) for uhRT (*p* = 0.72; log-rank *p* = 0.82). The HR for death following uhRT relative to nRT was 1.62 (95% CI 0.62–4.24, *p* = 0.33).

### 3.4. Wound Revision

A total of 9 (12.0%) of 74 patients in the nRT group and 8 (12.5%) of 64 patients treated with uhRT underwent wound revision (*p* = 0.42). The OR for uhRT compared to nRT was 0.80 (95% CI 0.24–2.59, *p* = 0.71).

## 4. Discussion

The present study is the first prospective observational study comparing uhRT and nRT for STS. Our results demonstrated no significant differences in LRFS, OS and wound complication rates between the uhRT group and the nRT group.

In this study, the two-year rates were 92% in the nRT group and 95% in the uhRT group, comparable with the rates reported in the current literature (Table 3) [7,10,25,33]. While nRT regimens, as reported in prospective studies by Canter et al. and Shah et al., achieved local control rates of approximately 100% at 3–5 years, they reported significant wound complication rates ranging from 23% to 35% [34,35]. Similarly, the phase III study of O’Sullivan et al. reported a 92% local control rate at 3.3 years with a 35% wound complication rate [5]. Two phase II studies of moderately and ultrahypofractionated schedules by Guadagnolo et al. and Bedi et al. also noted 31% and 25% wound complication rates, respectively [11,25]. The two existing phase II studies of ultrahypofractionated regimens demonstrated comparable results: Kosela et al. reported an 81% local control rate at 3 years with a 32% wound complication rate, while Kalbasi et al. achieved a 94% local control rate at 2 years with a 31% wound complication rate [14,15]. Our study validated the uhRT results in comparable local control rates to the nRT and hRT regimens. Notably, our study noted a wound complication rate of 12.5% in the uhRT group and 12% in the nRT group, significantly lower than previously reported rates after nRT and hRT, but consistent with our own historical control of an nRT cohort [6].

The evidence provided herein supports that uhRT may serve as a viable alternative treatment regimen for patients with STSs. A regimen with high therapeutic efficacy at a shorter treatment duration, such as uhRT, may reduce the treatment burden and encourage patient compliance. Additionally, the increased implementation of uhRT into clinical practice may lead to efficient resource allocation and reduced healthcare costs [15,25].

While prospective randomized trials are the gold standard, prospective real-world-time (RWT) data, collected during routine clinical practice, may mirror the treatment outcomes in diverse, real-world populations and provide more generalizable results. Furthermore, the prospective nature of RWT allows for the accurate and complete collection of follow-up data. A combination of machine learning and causal inference can help to infer individualized treatment effects [37,38].

Another interesting area of research involves nanoparticles which might be incorporated into a multimodal treatment regimen for sarcomas in the future [39,40].

This study has several limitations. The relatively small sample size and short follow-up period may limit the generalizability of the results. Although the cluster design and the lack of intersurgeon variability mitigate some biases, the non-randomized nature of treatment assignment remains a limitation. Future studies, including target trial emulations and randomized controlled trials, will be necessary to validate the long-term safety and efficacy of uhRT for the treatment of STSs. Furthermore, the follow-up time of the present study cohort was limited. Moreover, the quality of life of the included patients was not formally assessed. However, we plan to publish an extended follow-up and quality of life assessment in the future.

Our results indicate that uhRT, delivering 25 Gy in five fractions over one week, achieved similar local control, short-term overall survival rates and wound complication rates to nRT. The reduced treatment duration associated with uhRT offers substantial benefits in terms of patient convenience, compliance and healthcare costs. Therefore, uhRT may serve as an alternative radiotherapy regimen for the treatment of STSs. While our study provided level 2b evidence which encourages the adoption of uhRT for the treatment of STSs, larger randomized controlled studies with longer follow-up periods will be necessary to validate our results.

## 5. Conclusions

Our study presents uhRT as a promising alternative to nRT in the preoperative treatment of soft tissue sarcoma, with comparable rates of local control, survival and wound complications over the short term. The shorter duration of uhRT offers meaningful advantages for the patient experience and may improve healthcare resource utilization. However, the study’s non-randomized design and clustered approach warrant cautious interpretation.

These findings suggest that uhRT could be integrated as a patient-centered, efficient option in sarcoma care, aligning with the modern goals of value-based healthcare. Further randomized trials with extended follow-up are essential to confirm long-term outcomes and to refine the role of uhRT within sarcoma treatment protocols.

## Figures and Tables

**Table 1:** Definitions of major and minor wound complications.

Category	Definition/Examples
Major Wound Complications	Secondary operations required under general or regional anesthesia for wound treatment.
	Readmission to the hospital for wound care.
	Invasive procedures for wound management.
	Deep wound packing to an area of a wound measuring at least 2 cm in length.
	Prolonged dressing changes or repeat surgery for revision of a split-thickness skin graft.
	Wet dressings for longer than 4 weeks
Minor Wound Complications	Wound-edge necrosis requiring topical treatment such as silver sulfadiazine cream.
	Minor infections treated with oral antibiotics
	Prolonged dry dressing not reaching the extent or duration of “prolonged dressing changes” defined under major complications.

**Table 2:** Patient characteristics.

Characteristic	Overall, N = 138 ^1^	Normofractionation, N = 74 ^1^	Ultrahypofractionation, N = 64 ^1^	*p*-Value ^2^
Gender				0.83
Female	63 (46%)	33 (45%)	30 (47%)	
Male	75 (54%)	41 (55%)	34 (53%)	
Age	59 (49, 73)	59 (50, 74)	61 (49, 72)	0.90
Depth				0.31
Epifascial	22 (16%)	9 (12%)	13 (20%)	
Retroperitoneal	4 (2.9%)	3 (4.1%)	1 (1.6%)	
Subfascial	112 (81%)	62 (84%)	50 (78%)	
Size	83 (59, 120)	87 (65, 121)	74 (52, 110)	0.14
Grading				0.007
G1	13 (9.4%)	6 (8.1%)	7 (11%)	
G2	37 (27%)	27 (36%)	10 (16%)	
G3	72 (52%)	30 (41%)	42 (66%)	
(Unknown)	16 (12%)	11 (15%)	5 (7.8%)	
Necrosis	40 (10, 90)	60 (10, 90)	20 (5, 69)	0.002
Indication				0.11
First presentation	119 (86%)	67 (91%)	52 (81%)	
Recurrence	19 (14%)	7 (9.5%)	12 (19%)	
Hyperthermia	1 (1.0%)	1 (2.8%)	0 (0%)	0.4
Boost	2 (2.0%)	2 (5.6%)	0 (0%)	0.13
Predicted 5y OS using Sarculator or Persarc	0.78 (0.66, 0.88)	0.77 (0.63, 0.85)	0.82 (0.68, 0.90)	0.10
Status				0.40
AWD	32 (23%)	16 (22%)	16 (25%)	
DOD	31 (23%)	20 (27%)	11 (17%)	
NED	75 (54%)	38 (51%)	37 (58%)	
Wound revision	17 (12%)	9 (12%)	8 (13%)	0.93

^1^ n (%); Median (IQR). ^2^ Pearson’s Chi-squared test; Wilcoxon rank-sum test; Fisher’s exact test. Legend: AWD—alive with disease; DOD—dead of disease; NED—no evidence of disease.

**Table 3:** Characteristics of previous studies assessing different radiotherapy regimens.

Fractionation Type	Study	Design	Number of Patients	Total Dose	Dose/Fraction	Duration (Weeks)	Wound Complication Rate	Local Control
nRT	Canter et al. (2010) [36]	Prospective	25	50	2	5	28%	100% at 3-years
	Shah et al. (2012) [35]	Prospective	30	50	2	5	23%	100% at 5 years
	O’Sullivan et al. (2022) [5]	Phase III	94	50	2	5	35%	92% at 3.3 years
	Studer et al. (2018) [6]	Retrospective	67	50	2	5	7%	98% at 3 years
hRT	Guadagnolo et al. (2022) [25]	Phase II	120	42.75	2.85	3	31%	-
uhRT	Kosela-Paterczyk et al. (2016) [17]	Phase II	32	25	5	1	29%	90% at 5 years
	Kalbasi et al. (2020) [15]	Phase II	52	30	6	1	32%	94% at 2 years
	Bedi et al. (2022) [11]	Phase II	32	35	7	1	25%	100% at 3 years
	Present study (2024)	Comparative Phase II	64 (of 138)	25	5	1	12%	94.8% at 2.2 years

## Data Availability

The data presented in this study are available on request from the corresponding author.

## Supplementary Materials

The following supporting information can be downloaded at: https://www.mdpi.com/article/10.3390/cancers16234063/s1, Table S1: Diagnoses of included patients according to therapy group.
