# Supplementary material for: Ultrahypofractionated Versus Normofractionated Preoperative Radiotherapy for Soft Tissue Sarcoma: A Multicenter, Prospective Real-World-Time Phase 2 Clinical Trial
Source: Cancers (Basel). 2024 Dec 4;16(23):4063. doi: 10.3390/cancers16234063 (PMC11640059; doi:10.3390/cancers16234063)
Supplement: Supplementary file 1 [file cancers-16-04063-s001.zip › cancers-3337585-supplementary.pdf]

Supplementary Table S1. Diagnoses of included patients according to therapy group.

Characteristic	Overall N = 138 [1]	Standard Fractionation N = 74 [1]	Hypofractionation N = 64 [1]
Diagnosis			
Atypical lipomatous tumor (ALT)	1 (0.7%)	1 (1.4%)	0 (0%)
Alveolar rabdomyosarcoma	1 (0.7%)	1 (1.4%)	0 (0%)
Angiosarcoma	1 (0.7%)	1 (1.4%)	0 (0%)
Bifasic synovial sarcoma	1 (0.7%)	1 (1.4%)	0 (0%)
Chordoma	1 (0.7%)	1 (1.4%)	0 (0%)
Clear cell sarcoma	1 (0.7%)	0 (0%)	1 (1.6%)
Dedifferentiated liposarcoma	18 (13.0%)	10 (13.5%)	8 (12.5%)
Dedifferentiated liposarcoma with osteo and chondrosarcomatous tracts	1 (0.7%)	1 (1.4%)	0 (0%)
Dedifferentiated liposarcoma, inflammatory type	1 (0.7%)	1 (1.4%)	0 (0%)
Dedifferentiated liposarcoma, pleomorphic cell	1 (0.7%)	1 (1.4%)	0 (0%)
Epitelioid sclerotisig fibrosarcoma	1 (0.7%)	1 (1.4%)	0 (0%)
Epithelioid sarcoma	1 (0.7%)	0 (0%)	1 (1.6%)
Extraskeletal myxoid chondrosarcoma	1 (0.7%)	0 (0%)	1 (1.6%)
Extraskeletric myxoid chondrosarcoma	1 (0.7%)	1 (1.4%)	0 (0%)
High grade Myxofibrosarcoma	1 (0.7%)	1 (1.4%)	0 (0%)
High grade spindle cell	1 (0.7%)	0 (0%)	1 (1.6%)
Leiomyosarcoma	8 (5.8%)	3 (4.1%)	5 (7.8%)
Liposarcoma	1 (0.7%)	1 (1.4%)	0 (0%)
Low grade fibromyxoid saroma (Evans tumor)	1 (0.7%)	1 (1.4%)	0 (0%)
Monophasic synovial sarcoma	1 (0.7%)	1 (1.4%)	0 (0%)
Malignant peripheral nerve sheath tumor	4 (2.9%)	3 (4.1%)	1 (1.6%)
Myxofibrosarcoma	20 (14.5%)	6 (8.1%)	14 (22%)
Myxoid chondrosarcoma	1 (0.7%)	0 (0%)	1 (1.6%)
Myxoid liposarcoma	14 (10.1%)	6 (8.1%)	8 (13%)
Partially dediff., partially myogenous, soft tissue sarcoma	1 (0.7%)	1 (1.4%)	0 (0%)
Pleomorphic liposarcoma	5 (3.6%)	4 (5.4%)	1 (1.6%)
Pleomorphic rabdomyosarcom	1 (0.7%)	1 (1.4%)	0 (0%)
Pleomorphic sarcoma	1 (0.7%)	1 (1.4%)	0 (0%)
Pleomorphic, spindle cell, sarcoma	1 (0.7%)	1 (1.4%)	0 (0%)
Sclerotizing epithelioid fibrosarcoma	2 (1.4%)	2 (2.7%)	0 (0%)
Spindle cell, pleomorphic sarcoma	1 (0.7%)	1 (1.4%)	0 (0%)
Synovial sarcoma	4 (2.9%)	3 (4.1%)	1 (1.6%)
Undifferentiated pleomorphic sarcoma	37 (27%)	16 (22%)	21 (33%)
Undifferentiated pleomorphic sarcoma, high grade	1 (0.7%)	1 (1.4%)	0 (0%)
Vascular leiomyosarcoma	1 (0.7%)	1 (1.4%)	0 (0%)

[1] n (%).
